# Insights Into the Ecomorphology of the Blue Shark ( *Prionace glauca* ) in the Adriatic Sea (Central Mediterranean Sea)

**DOI:** 10.1002/ece3.72478

**Published:** 2025-11-09

**Authors:** P. Carbonara, A. Bellodi, M. Bottaro, G. Deplano, A. Mulas, C. Neglia, S. Niedermüller, G. Prato, L. Toomey, M. C. Follesa

**Affiliations:** ^1^ Fondazione COISPA ETS Bari Italy; ^2^ Department of Integrated Marine Ecology Calabria Marine Center, Stazione Zoologica Anton Dohrn (SZN) Amendolara Italy; ^3^ Department of Life and Environmental Sciences University of Cagliari Cagliari Italy; ^4^ Department of Integrative Marine Ecology Genoa Marine Centre, Stazione Zoologica Anton Dohrn – Italian National Institute of Marine Biology, Ecology and Biotechnology Genoa Italy; ^5^ WWF‐Mediterranean Rome Italy; ^6^ WWF‐Italia Rome Italy; ^7^ CoNISMa Consorzio Nazionale Interuniversitario per le Scienze Mare Rome Italy

**Keywords:** blue shark, functional morphology, linear morphometry, morphometry, ontogenetic development, pelagic shark, *Prionace glauca*

## Abstract

The blue shark ( *Prionace glauca* ) is a widely distributed pelagic predator that undergoes ontogenetic morphological changes that reflect ecological and functional adaptations. Investigating this aspect could provide powerful tools for elucidating the patterns of trait variation and their significance in relation to environmental conditions. This study explores morphometric variation and its ecomorphological implications in the Mediterranean blue shark population using linear and geometric morphometric analyses. A total of 119 specimens (64 females, 55 males), spanning from juvenile (total lengths [TLs] 85.4–149.8 cm in females and 82.9–121.2 cm in males) to adult (TLs 150.4–333.1 cm in females and 133.2–206.8 cm in males) stages, were analysed through a set of 30 linear measurements. Linear morphometric analysis identified significant differences between the juvenile and subadult/adult groups, particularly in the anterior body region and pectoral fin measurements. Geometric morphometric results revealed that juveniles have a more heterocercal caudal fin shape, which transitions to a less heterocercal form in adults, with a proportionally larger ventral lobe. The first dorsal fin showed positive allometry, becoming higher. The observed ontogenetic differences could improve energy efficiency and manoeuvrability. This aligns with the diel vertical migrations (DVM) of larger individuals and their need for sustained swimming during long‐distance migrations, as well as the enhanced manoeuvrability required by juveniles, notably for predator avoidance. These morphological changes likely reflect adaptations to changing energy requirements and locomotion needs throughout life stages. Although blue sharks maintain a generalist diet across ontogeny, these adaptations may optimise feeding efficiency and swimming performance, particularly in adults. This study highlights the interplay between ontogeny, morphology and ecological function, shedding light on the selective pressures that shape the life history strategies of blue sharks. The findings emphasise the importance of integrating ontogenetic perspectives into ecomorphological studies to better understand the evolutionary adaptations of pelagic sharks.

## Graphical abstract

This study examines how the morphology of Mediterranean blue sharks ( *Prionace glauca* ) changes throughout their development. Using linear and geometric morphometrics, it reveals that juveniles and adults differ in fin and head shape, reflecting adaptations to different ecological and swimming demands. These ontogenetic changes likely enhance energy efficiency, manoeuvrability and overall performance across life stages.

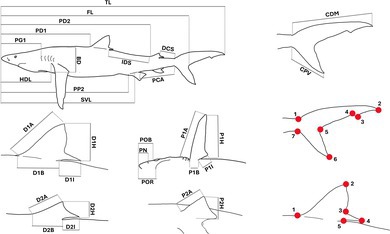

## 1 Introduction

The blue shark ( *Prionace glauca* L. 1758) is one of the most abundant and widely distributed pelagic shark species, inhabiting the tropical to temperate regions of the Atlantic and Indo‐Pacific oceans, as well as the Mediterranean Sea (Druon et al. 2022 and references within). Because of its widespread distribution, the blue shark is also a key species in the global shark meat and fin trade, being one of the most commonly captured and landed pelagic shark species worldwide (Gilman et al. 2008; Eriksson and Clarke 2015; Cordova‐Zavaleta et al. 2018; Niedermüller et al. 2021). This species can reach lengths of around 400 cm and weights exceeding 200 kg, starting at approximately 30 cm in length (1 kg total mass) at birth (Kohler et al. 2002; Ebert et al. 2021). The blue shark is a generalist/opportunistic predator (Loor‐Andrade et al. 2017; Bazzi et al. 2021 and references therein) with a relatively constant diet throughout its life (Rabehagasoa et al. 2012; Vandeperre et al. 2014; Hernández‐Aguilar et al. 2016). However, despite maintaining a generalist diet, its somatic growth may enable the blue shark to feed on larger and more agile prey. The blue shark performs frequent upward and downward dives (Queiroz et al. 2012; Vedor et al. 2021; Druon et al. 2022), diving from the ocean surface to depths of up to 1500 m (Queiroz et al. 2012). This behaviour has been observed in several locations, including the Atlantic Ocean (Queiroz et al. 2012; Vedor et al. 2021) and the Mediterranean Sea, and in both juvenile and adult specimens (Carbonara et al. 2024). These vertical migrations are believed to improve foraging success (Campana et al. 2011; Bandara et al. 2021) by increasing spatial overlap with prey or by actively chasing prey's vertical movements (e.g., Braun et al. 2023). This species also exhibits significant long‐range spatial migrations, including within the Mediterranean Sea (Poisson et al. 2024). The Mediterranean population is currently managed as a single stock, distinct from the North Atlantic population. However, recent evidence suggests the existence of substructuring within the Mediterranean Sea. This is demonstrated by the genetic differentiation observed between Western and Eastern Mediterranean blue sharks (Leone et al. 2024). This is also supported by the limited connectivity indicated by telemetry data (Poisson et al. 2024).

The International Union for Conservation of Nature (IUCN) has classified the Mediterranean blue shark population as “critically endangered” (Sims and Queiroz 2016), whereas the global population is listed as “near threatened” (Rigby et al. 2019). Fishing mortality, both from targeted fisheries and as unwanted bycatch in fisheries that target other fish species, is the primary driver of the blue shark population decline (Tsai and Liu 2015; Campana et al. 2016).

Sharks are a morphologically diverse and evolutionarily successful group of vertebrates, with over 500 species adapted to a wide range of marine environments (Sternes and Shimada 2020). Their eco‐morphological diversity reflects both long‐term evolutionary pressures and developmental (ontogenetic) changes, often influenced by habitat use and ecological shifts such as diet. Understanding these morphological patterns provides valuable insight into the evolutionary drivers shaping shark biodiversity (Sternes and Shimada 2020; Bazzi et al. 2021; López‐Romero et al. 2023; Gayford et al. 2025).

Body shape is a key aspect of an organism's phenotype, influencing essential life functions such as movement, hunting, evading predators, migration and reproductive success (Wainwright 1991; Bock 1994). Ecomorphological studies, which seek to understand the morphological adaptations of organisms to specific habitats, therefore focus on body shape. This phenotype is shaped not only by developmental and phylogenetic constraints, but also by evolutionary selection (e.g., Sternes and Shimada 2020).

Studies of the morphometric characteristics of elasmobranchs are among the most powerful tools for elucidating the evolutionary trajectory and significance of particular traits in relation to their environments (Motani 2002, 2005; Sternes and Shimada 2020). Moreover, morphometric analysis is regarded as a powerful and cost‐effective method to identify cryptic species or the presence of different subpopulations within the same species (Quattro et al. 2013; Chaklader et al. 2016; Carbonara et al. 2021). Indeed, it has long been recognised that adaptive morphological variations occur not only at the species level, but also at the intraspecific level, for example, between populations and different developmental stages (Pélabon et al. 2013; Pèlabon et al. 2014). Growth is associated not only with an increase in body size, but also with functional adaptations in traits related to ontogenetic shifts in habitat use and/or diet (Gayford, Godfrey, and Whitehead 2023; Gayford, Whitehead, et al. 2023). Therefore, ontogenetic changes in body size and/or shape may reflect the selective pressures that drive morphometric scaling in the morphology of elasmobranchs, such as sharks (Gayford, Godfrey, and Whitehead 2023; Gayford, Whitehead, et al. 2023). From this perspective, ontogenetic changes in diet and habitat represent important drivers of morphometric scaling, as summarised by Gayford, Whitehead, et al. (2023) in the allometric niche shift hypothesis.

Morphological variations during developmental stages have been observed in several shark species (e.g., Lingham‐Soliar 2005; Lowry et al. 2007; Irschick and Hammerschlag 2015; Wilga et al. 2016; Gleiss et al. 2017; Irschick et al. 2017; Natanson et al. 2018; Ahnelt et al. 2020; Sternes and Higham 2022; Goodman et al. 2022; Bellodi et al. 2023; Yun and Watanabe 2023; Gayford, Godfrey, and Whitehead 2023; Gayford, Whitehead, et al. 2023; Gayford, Whitehead, and Jaquemet 2024; Gayford, Sternes, et al. 2024; Gayford, Waghe, et al. 2024; Hunt et al. 2025), supporting the hypothesis that allometric niche shift underlies ontogenetic scaling. Seamone et al. (2024) have shown that the blue shark exhibits significant morphological adaptations and/or changes throughout ontogenetic development, but this work was limited to sharks caught in the Atlantic Ocean (off the coast of Nova Scotia, Canada). However, given the extremely low connectivity between Atlantic and Mediterranean blue shark populations (Leone et al. 2024; Poisson et al. 2024), differences in body scaling may exist between the two areas. This emphasises the importance of conducting morphometric studies that are specific to the Mediterranean Sea. Furthermore, Carbonara et al. (2024) recently demonstrated that larger blue sharks are capable of reaching greater depths. Therefore, the need for an effective strategy to facilitate wider vertical migrations could be a key factor in body development during ontogeny.

The present study is the first to examine the ontogenetic morphometric characteristics of Mediterranean blue sharks. It analyses morphological data from a large number of individuals across three size classes (i.e., juveniles, subadults, and adults), covering a wide length range, which may reveal morphometric trends associated with distinct life stages. The research includes a detailed geometric morphometric analysis of the caudal and dorsal fins, combined with a linear morphometric analysis of the whole shark body. In order to produce precise and interpretable results, the data were grouped by sex and life stage, facilitating comparisons with existing ecomorphological and trophic/spatial ecology findings. Such an approach highlights how ontogenetic differences may optimise vertical migrations or favour feeding on different prey types (Blaison et al. 2015; Fu et al. 2016; Franks et al. 2021; Rider et al. 2021), in what is considered the most threatened blue shark population worldwide (Sims and Queiroz 2016). Observed allometric changes provide insights into behavioural and functional aspects during ontogeny, illustrating how ecomorphological methods can yield valuable information on the life history of large pelagic shark species such as the blue shark, which are often challenging to study in their natural environments.

## 2 Materials and Methods

### 2.1 Sampling

Between 2019 and 2024, a total of 119 blue shark specimens were caught as part of the routine monitoring of the commercial pelagic longline fishery and the SafeShark (WWF 2022) and MedByCatch (https://medasset.org/portfolio‐item/medbycatch‐project/) tagging projects in the Adriatic Sea (FAO Geographic Sub‐Area 18) (see Carbonara et al. 2024). The sharks were caught using longlines (approximately 30–40 km long), each of which was equipped with a 13 m long dropline at about every 58 m intervals. These droplines carried a 76 mm J type hook, which, once in the fishing position, lay about 30 m below the sea surface (Dapp et al. 2016; Carbonara et al. 2023). Longlines were deployed overnight for 10–20 h. Upon retrieval, measurements were conducted either onboard, where living specimens were released back into the water within a maximum retention time of 4–8 min, following the procedures described by Carbonara et al. (2024), or at the landing site, when dead specimens were brought to the harbour. For each specimen caught, TL (in cm) and sex were recorded.

### 2.2 Linear Morphometry

**FIGURE 1:**
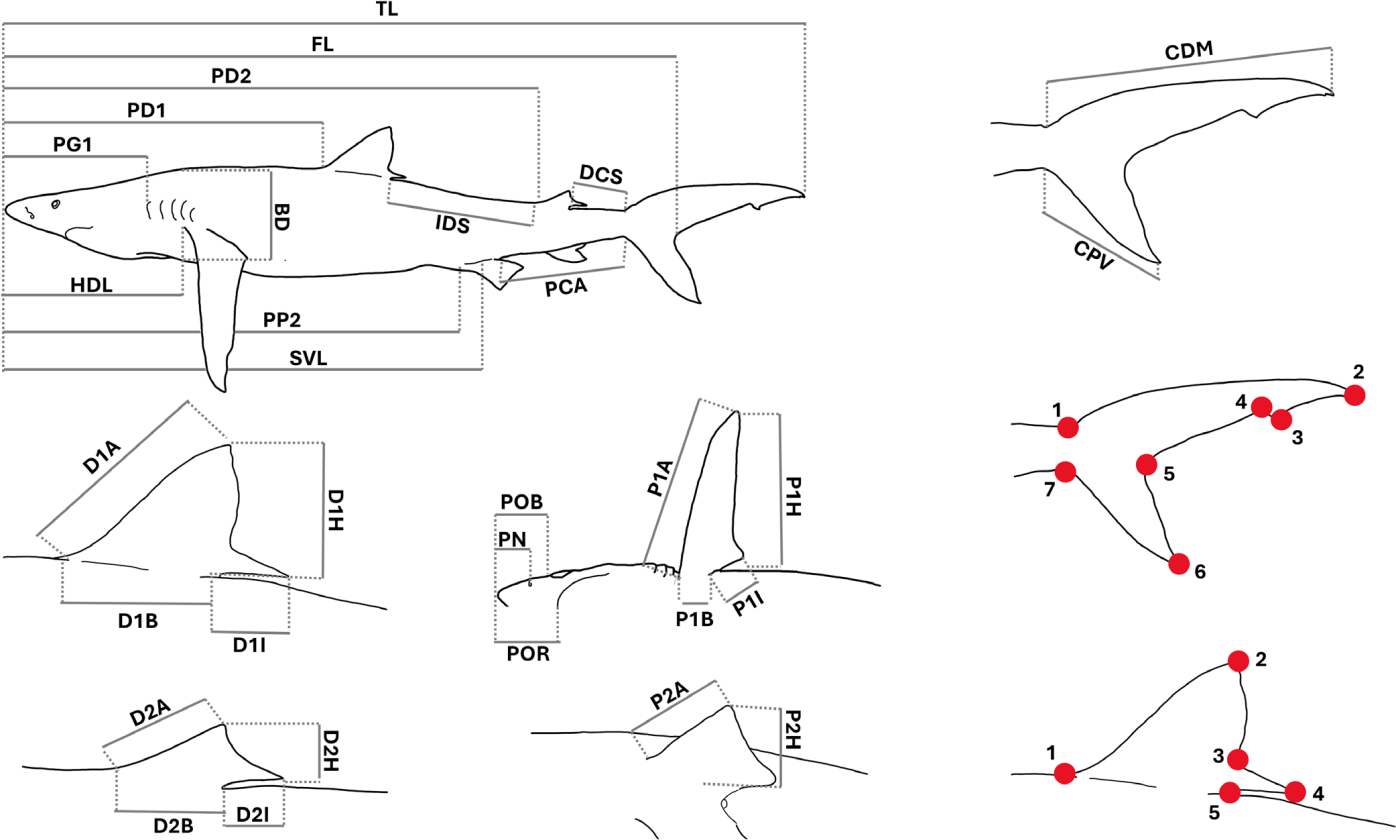
Linear morphometric measurements, with the relative acronyms defined in Table 1, and position of the landmarks (red dots) placed on the caudal and first dorsal fins of *Prionace glauca* .

Either directly on board or at the landing site, lateral and ventral pictures together with specific images of each fin (Figure 1), were taken of every blue shark, with a high‐definition camera (Canon 650D equipped with a Canon 18‐55 lens). To minimise the effects of optical distortion, each photograph was taken with a focal length of 40 mm and the camera lens was positioned perpendicular to the specimen, as verified using a camera‐mounted spirit level. The distance between the lens and the subject varied according to the size of the shark or the body region of interest, and a scale was included in each image. The obtained images were used to record 30 different linear measurements (Table 1; Figure 1) (Compagno 2001; Last et al. 2007; Bellodi et al. 2023) using the TPSDig.2 v 2.31 software (Rohlf 2005). To identify scaling relationships, all measurements were expressed as a percentage of TL (Bellodi et al. 2022, 2023). For dead specimens brought to the landing site, the accuracy of the photographic measurements was verified by comparing them with direct measurements of TL, pre‐ventral length, dorsal and pectoral fin heights, and the caudal dorsal margin, all of which were recorded on the same individuals. All specimens were grouped into three size classes on the basis of TL at first maturity (Megalofonou et al. 2009) and, when possible (dead specimens landed and adult/subadult male specimens released live through claspers' observation), on the basis of direct gonad analysis (Follesa and Carbonara 2019). The groups were defined as follows: (i) juveniles (J), comprising females and males with TLs inferior to 150 and 130 cm, respectively; (ii) subadults (SA), comprising females with TLs between 150 and 180 cm and males with TLs between 130 and 150 cm; and (iii) adults (A), comprising specimens with TLs superior to 150 cm for males and 180 cm for females.

**TABLE 1:** Acronyms and brief descriptions of each linear measurement recorded in *Prionace glauca* .

Acronym	Description	Acronym	Description
FL	Fork length	POR	Pre‐oral length
PD1	Pre‐first dorsal fin length	D1A	First dorsal fin anterior margin
PD2	Pre‐second dorsal fin length	D1B	First dorsal fin base length
PG1	Pre‐branchial length	DIH	First dorsal fin height
BD	Body diameter at the origin of the pectoral fin	D1I	First dorsal fin inner margin
IDS	Interdorsal space	D2A	Second dorsal fin anterior margin
DCS	Dorsal caudal fin space	D2B	Second dorsal fin base length
CDM	Dorsal caudal fin margin	D2H	Second dorsal fin height
PCA	Pelvic fin caudal fin space	D2I	Second dorsal fin inner margin
CPV	Pre‐ventral caudal fin margin	P1A	Pectoral fin anterior margin
HDL	Head length	P1B	Pectoral fin base
PP2	Pre‐pelvic fin length	P1H	Pectoral fin height
SVL	Snout‐vent length	P1I	Pectoral fin inner margin
POB	Preorbital length	P2A	Pelvic fin anterior margin
PN	Prenostrils length	P2H	Pelvic fin height

Given that sexual dimorphism appears to be particularly prevalent among elasmobranchs (e.g., Kajiura et al. 2005; Orlov and Cotton 2011; Barbosa Martins et al. 2015; Gayford 2023; Gayford and Sternes 2024), a Canonical Analysis of Principal Coordinates (CAP) was initially conducted on specimens grouped by sex to evaluate whether sex should be considered a factor when investigating the allometric growth of blue sharks. The CAP analysis was performed using the PRIMER v.7 software (Clarke and Gorley 2015) on a similarity matrix on the basis of Euclidean distances. When no differences between the sexes were found, specimens of both sexes were grouped to examine the impact of size on body proportions. After the assessment of sex as a factor, a second CAP analysis was conducted on specimens grouped by size class (J, SA, A) to investigate changes in body proportions during ontogenetic growth. In this context, the CAP procedure also provides a measure of misclassification, defined as the proportion of specimens incorrectly assigned to groups relative to their a priori classification (male or female, or J, SA or A). Lower misclassification rates indicate stronger separation between groups, whereas higher rates suggest greater overlap in body proportions. Furthermore, a principal component analysis (PCA) was conducted in the R environment (v. 4.4.1; R Core Team 2022) using the ggbiplot R package (v. 0.6.2; Vu et al. 2024) on a correlation matrix of the same linear measurements, to corroborate the CAP results for both sex and size class groupings. Within the same software, a multivariate analysis of variance (MANOVA) on the basis of Pillai's test (Pillai 2014) was also conducted to test the statistical significance of the first three principal components in each analysis. Finally, to determine which morphometric measurements were most important in defining size groups, a Similarity Percentage (SIMPER) analysis was carried out using the PRIMER v.7 software.

Finally, to test whether each of these measurements scaled isometrically with respect to blue shark TL, all measurements, including TL, were log_10_‐tranformed in order to ensure linearity; the scaling equation was: log_10_(*y*) = log_10_(*a*) + *b*log_10_(TL) (Higham et al. 2018; Seamone et al. 2024; Hunt et al. 2025). Length measurements that scale isometrically with other lengths (TL) are expected to show scaling exponent values close to 1. The 95% confidence intervals (CI) and standard errors of the scaling factor, along with the *R* ^2^ value of the regression, were calculated. Isometry was assessed using a *t*‐test. If *p* > 0.05, the measurement was considered to grow isometrically with respect to TL. If the scaling factor was significantly greater than 1, the measurement was classified as showing positive allometry; if it was significantly less than 1, it was classified as showing negative allometry.

### 2.3 Fin's Morphometry

A geometric morphometric analysis of blue shark fins was conducted on a subsample of 64 specimens for the caudal fin and 26 specimens for the first dorsal fin. This reduction in sample size was mainly due to the work being carried out on board with live animals that had to be released afterwards. In fact, these animals often moved, which meant that some photographs of the fins, although suitable for recording linear measurements (which could frequently be cross‐checked with the same measurements taken on the same structure from lateral photos of the whole individual), were unusable for landmark placement. This occurred either because of slight movements, the fin not being in the standardised position required for the analysis, or because of highly variable lighting conditions (including reflections on the wet skin of the animal) on board the fishing vessel. Furthermore, all operations were carried out with the aim of promptly releasing the animals, as they could not be kept out of the water for extended periods. For this reason, in order to ensure the highest possible precision in the analysis, we chose to exclude all photographs that were not of optimal quality. Using the TPSDig2 software, a total of seven landmarks were placed on each caudal fin image, whereas five landmarks were set on the anterior dorsal fins. The landmarks were chosen for their easily detectable position (Figure 1). The geometric morphometric analysis was performed using the MorphoJ software (Klingenberg 2011). The first step consisted of performing a Procrustes transformation on the TPS file containing the landmark coordinates to align principal axes and scale the pictures. This ultimately allowed an unbiased shape comparison. A discriminant function analysis (DFA) was performed for evaluating the caudal and dorsal fin shapes of blue sharks grouped by sex and then by size (J, SA, A). This produced a wireframe graph to display results. Reported *p*‐values were adjusted using the Bonferroni correction. Finally, using the same software, another PCA, along with the shape changes graph, was performed in order to understand how much of the variation is explained and how the fin shape changes during ontogeny. Although this approach, which relies solely on landmarks, captures the general fin shape effectively, it may fail to identify subtle intergroup shape differences that warrant investigation through the use of semi‐landmarks.

## 3 Results

### 3.1 Sample Composition

During sampling, a total of 64 female and 55 male blue sharks were caught. The females ranged in TL from 85.4 to 333.1 cm, whereas the males ranged from 82.9 to 206.8 cm. Further details on the sample composition, divided by sex and size groups, are reported in Table 2. Linear relationship data of each measurement in relation to TL are available as Table S1.

**TABLE 2:** *Prionace glauca* sampled individuals.

	Size group	*N* specimens	TL (cm) range	Mean TL (cm) ± SD
Females	J	31	85.4–149.8	130.0 ± 15.2
	SA	22	150.4–174.7	160.5 ± 8.2
	A	11	180.9–333.1	211.2 ± 45.4
Males	J	5	82.9–121.2	102.5 ± 17.4
	SA	20	133.2–149.7	142.8 ± 5.1
	A	30	151.1–206.8	169.8 ± 16.3

Abbreviations: A, adults; J, juveniles; SA, subadults; SD, standard deviation; TL, total length.

### 3.2 Linear Morphometry

**FIGURE 2:**
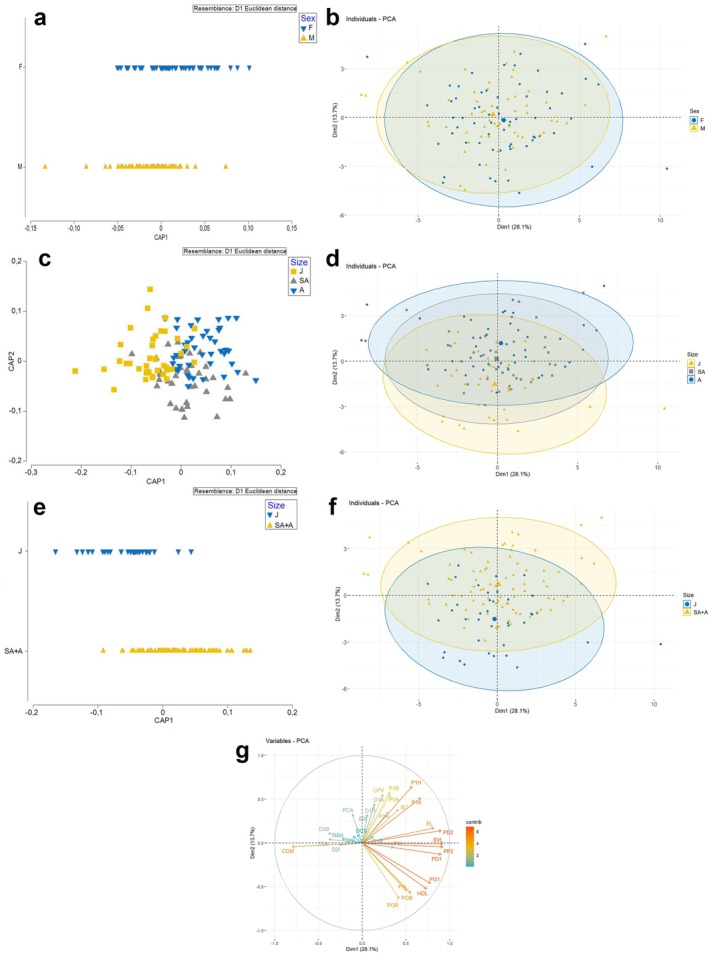
CAP (left) and PCA (right; with 70% confidence ellipses) plots on the basis of linear morphometric measurements in *Prionace glauca* specimens grouped by sex (a, b; F, females; M, males), by size (A, adults; J, juveniles; SA, subadults; c, d) and comparing juveniles against subadults and adults combined (e, f). The contribution of each linear measurement is also shown in g.

The first CAP analysis, conducted using the specimen's sex as a factor, returned an overall misclassification error of 36.13%. Specifically, males were correctly grouped in 34 out of 55 cases (61.8%), whereas females were in 42 out of 62 cases (65.6%) (Table 3). Both the CAP (Figure 2a) and PCA (Figure 2b) analyses showed a substantial overlap between the sexes. These results were further confirmed by the MANOVA test, which failed to detect statistical differences between the sexes in the first three principal components (Pr > *F*; 0.1826).

**TABLE 3:** Summary of the CAP results on the basis of linear morphometric measurements of *Prionace glauca* using sex (F, females; M, males), size (A, adults; J, juveniles; SA, subadults) and a combined grouping of subadults and adults. The table shows the correlation and correlation‐squared (Corr. Sq.) values, the overall percentage of misclassification, how each a priori classified specimen (Orig. Group) was grouped by the analysis (Computed group) and the percentage of correct classification.

Factor	Correlation	Corr. sq.	Misclassification error (%)	Orig. group	Computed group			Total	% Correct
					F	M	—		
Sex	0.3846	0.1479	36.13	F	42	22	—	62	65.63
				M	21	34	—	55	61.82

Factor	Correlation	Corr. sq.	Misclassification error (%)	Orig. group	Computed group			Total	% Correct
					J	SA	A		
Size	0.6858	0.4703	35.29	J	28	7	3	38	73.68
				SA	3	21	14	38	55.26
				A	2	13	28	43	65.12

Factor	Correlation	Corr. sq.	Misclassification error (%)	Orig. group	Computed group			Total	% Correct
					J	SA + A	—		
Size subadults and adults united	0.6354	0.4037	18.49	J	31	7	—	38	81.57
				SA + A	15	66	—	81	81.48

In contrast, the CAP analysis carried out on size groups (J, SA, A) produced a similar overall misclassification value to that for sexes (35.3%; Table 3), but was effective in correctly distinguishing between juveniles (correctly classified in 28 out of 38 cases; 73.7%) and adults (correctly classified in 28 out of 43 cases; 65.1%). Subadults emerged as the group with the highest rate of misclassification, being correctly classified in only 21 out of 38 cases (55.3%) and frequently (14 times) misclassified as adults (Table 3). Nevertheless, both the CAP (Figure 2c) and PCA (Figure 2d) plots showed less overlap between groups than the sex analysis, as confirmed by the MANOVA test, which revealed statistically significant differences (Pr < *F*; < 0.001).

The high misclassification degree between subadults and adults (Table 3) suggested that these two groups may not constitute two distinct morphological groups. To test this hypothesis, we conducted an additional analysis in which we grouped adults and subadults together and compared them against juveniles. The CAP analysis performed better with this grouping as it returned a substantially lower misclassification error rate (18.5%; Table 3). As a result, both groups showed higher rates of correct identification: juveniles were correctly classified in 31 out of 38 cases (81.6%; Table 3), whereas the group composed of adults and subadults was correctly classified in 66 out of 81 cases (81.5%; Table 3). Both the CAP (Figure 2e) and PCA (Figure 2f) plots showed a low degree of overlap between the groups. Additionally, the MANOVA test revealed statistically significant differences between groups (Pr < *F*; < 0.001), which further supports this separation.

Finally, the SIMPER analysis (Table S2) was implemented to identify the most important measurements for discriminating between juveniles and the combined adult and subadult group. According to this analysis, the pectoral fin height (P1H) and the anterior margin (P1A) were the first and third most important measurements, accounting for 5.92% and 4.4% of the average dissimilarity between the groups, respectively. The second most important measurement for discriminating between the groups was the body diameter (BD), contributing 5.56%. Finally, the head length (HDL) and, a very similar measurement, the pre‐branchial length (PG1), each accounted for 3.8% of the dissimilarity. Together, these five measurements, which are mostly related to the head and pectoral fin regions, accounted for almost a quarter of the total dissimilarity between groups (23.47%) (Table S2).

In consideration of the CAP and PCA analysis results, which showed a better group separation between juveniles and subadults–adults together, the scaling relationship analysis was only carried out on these groups (Table 4). Among juveniles, most morphometric measurements exhibited isometric scaling. The only exceptions were the caudal ventral margin (CPV) and the anterior margin (P1A), base (P1B), and height (P1H) of the pectoral fin, all of which displayed positive allometric growth (Table 4). The only measurement that showed negative allometry in juveniles was the pre‐oral length (Table 4). In the subadult and adult group, the dorsal–caudal space (DCS) and the caudal fin dorsal margin (CDM) exhibited negative allometry (Table 4), whereas the pre‐second dorsal fin length (PD2), pre‐ventral fin length (PP2), snout–ventral length (SVL), BD and first dorsal fin height (D1H) displayed positive allometry (Table 4). Moreover, as observed in juveniles, the pectoral fin's anterior margin (P1A), base (P1B) and height (P1H) continued to exhibit positive allometry (Table 4).

**TABLE 4:** Scaling relationships obtained from linear regressions between log_10_‐transformed morphological variables and log_10_‐transformed total length (TL) in *Prionace glauca* .

Size group	Equation	*a*	*b*	*b* ± CI	SE slope	Adj *R* ^2^	*p*	Scaling
Juveniles	log_10_FL = log_10_ *a* + *b*log_10_TL	−0.1249	1.0207	0.0658	0.0324	0.9639	0.5270	Isometry
	log_10_PD2 = log_10_ *a* + *b*log_10_TL	−0.2531	1.0314	0.0812	0.0400	0.9471	0.4379	Isometry
	log_10_PD1 = log_10_ *a* + *b*log_10_TL	−0.4623	1.0233	0.1021	0.0503	0.9176	0.6467	Isometry
	log_10_PG1 = log_10_ *a* + *b*log_10_TL	−0.5653	0.9176	0.1871	0.0923	0.7258	0.3778	Isometry
	log_10_BD = log_10_ *a* + *b*log_10_TL	−0.8799	0.9829	0.3516	0.1734	0.4571	0.9221	Isometry
	log_10_IDS = log_10_ *a* + *b*log_10_TL	−0.4875	0.9375	0.1208	0.0595	0.8697	0.3006	Isometry
	log_10_DCS = log_10_ *a* + *b*log_10_TL	−0.9161	0.8965	0.2518	0.1242	0.5802	0.4100	Isometry
	log_10_CDM = log_10_ *a* + *b*log_10_TL	−0.5170	0.9553	0.1242	0.0612	0.8675	0.4697	Isometry
	log_10_PCA = log_10_ *a* + *b*log_10_TL	−0.6733	0.9772	0.1500	0.0739	0.8244	0.7591	Isometry
	log_10_CPV = log_10_ *a* + *b*log_10_TL	−1.4087	1.2322	0.2025	0.0998	0.8035	0.0258	Positive allometry
	log_10_HDL = log_10_ *a* + *b*log_10_TL	−0.4232	0.8856	0.1765	0.0870	0.7348	0.1971	Isometry
	log_10_PP2 = log_10_ *a* + *b*log_10_TL	−0.3291	1.0242	0.0987	0.0487	0.9227	0.6214	Isometry
	log_10_SVL = log_10_ *a* + *b*log_10_TL	−0.3232	1.0283	0.0931	0.0459	0.9312	0.5417	Isometry
	log_10_POB = log_10_ *a* + *b*log_10_TL	−0.6374	0.7399	0.2876	0.1418	0.4148	0.0749	Isometry
	log_10_PN = log_10_ *a* + *b*log_10_TL	−1.0528	0.8184	0.2880	0.1420	0.4655	0.2092	Isometry
	log_10_POR = log_10_ *a* + *b*log_10_TL	−0.4842	0.6999	0.2816	0.1388	0.3976	0.0374	Negative allometry
	log_10_D1A = log_10_ *a* + *b*log_10_TL	−1.2749	1.1100	0.2155	0.1063	0.7450	0.3077	Isometry
	log_10_D1B = log_10_ *a* + *b*log_10_TL	−1.0961	0.9678	0.2159	0.1064	0.6882	0.7643	Isometry
	log_10_D1H = log_10_ *a* + *b*log_10_TL	−1.8112	1.2663	0.2979	0.1469	0.6647	0.0781	Isometry
	log_10_D1I = log_10_ *a* + *b*log_10_TL	−0.8320	0.6517	0.5366	0.2646	0.1204	0.1964	Isometry
	log_10_D2A = log_10_ *a* + *b*log_10_TL	−0.7897	0.6904	0.4062	0.2003	0.2273	0.1309	Isometry
	log_10_D2B = log_10_ *a* + *b*log_10_TL	−0.8485	0.7245	0.3642	0.1796	0.2923	0.1337	Isometry
	log_10_D2H = log_10_ *a* + *b*log_10_TL	−0.9274	0.6054	0.4519	0.2228	0.1471	0.0851	Isometry
	log_10_D2I = log_10_ *a* + *b*log_10_TL	−1.1927	0.7363	0.6290	0.3101	0.1113	0.4007	Isometry
	log_10_P1A = log_10_ *a* + *b*log_10_TL	−1.4576	1.3548	0.1523	0.0751	0.8977	0.0000	Positive allometry
	log_10_P1B = log_10_ *a* + *b*log_10_TL	−2.2386	1.4443	0.3044	0.1501	0.7123	0.0054	Positive allometry
	log_10_P1H = log_10_ *a* + *b*log_10_TL	−1.9582	1.5306	0.2643	0.1303	0.7873	0.0002	Positive allometry
	log_10_P1I = log_10_ *a* + *b*log_10_TL	−1.5601	1.0884	0.2564	0.1264	0.6639	0.4889	Isometry
	log_10_P2A = log_10_ *a* + *b*log_10_TL	−1.8013	1.2592	0.2599	0.1281	0.7209	0.0505	Isometry
	log_10_P2H = log_10_ *a* + *b*log_10_TL	−2.0425	1.2737	0.3559	0.1755	0.5827	0.1276	Isometry
Subadults + adults	log_10_FL = log_10_ *a* + *b*log_10_TL	−0.1150	1.0164	0.0532	0.0267	0.9475	0.5418	Isometry
	log_10_PD2 = log_10_ *a* + *b*log_10_TL	−0.3674	1.0836	0.0596	0.0300	0.9423	0.0066	Positive allometry
	log_10_PD1 = log_10_ *a* + *b*log_10_TL	−0.5735	1.0695	0.1020	0.0513	0.8445	0.1792	Isometry
	log_10_PG1 = log_10_ *a* + *b*log_10_TL	−0.8472	1.0398	0.1779	0.0894	0.6267	0.6571	Isometry
	log_10_BD = log_10_ *a* + *b*log_10_TL	−1.9941	1.4921	0.2642	0.1327	0.6105	0.0004	Positive allometry
	log_10_IDS = log_10_ *a* + *b*log_10_TL	−0.6506	1.0161	0.0778	0.0391	0.8940	0.6820	Isometry
	log_10_DCS = log_10_ *a* + *b*log_10_TL	−0.6380	0.7856	0.1556	0.0782	0.5556	0.0075	Negative allometry

	log_10_CDM = log_10_ *a* + *b*log_10_TL	−0.2883	0.8501	0.1404	0.0706	0.6431	0.0367	Negative allometry
	log_10_PCA = log_10_ *a* + *b*log_10_TL	−0.7424	1.0157	0.1024	0.0514	0.8293	0.7617	Isometry
	log_10_CPV = log_10_ *a* + *b*log_10_TL	−1.1658	1.1188	0.1598	0.0803	0.7072	0.1429	Isometry
	log_10_HDL = log_10_ *a* + *b*log_10_TL	−0.7263	1.0198	0.1361	0.0684	0.7345	0.7734	Isometry
	log_10_PP2 = log_10_ *a* + *b*log_10_TL	−0.4821	1.0917	0.0851	0.0428	0.8905	0.0352	Positive allometry
	log_10_SVL = log_10_ *a* + *b*log_10_TL	−0.4563	1.0863	0.0823	0.0414	0.8960	0.0401	Positive allometry
	log_10_POB = log*a* + log*b*TL	−1.2462	1.0152	0.2003	0.1006	0.5575	0.8804	Isometry
	log_10_PN = log_10_ *a* + *b*log_10_TL	−1.3268	0.9370	0.2361	0.1186	0.4342	0.5971	Isometry
	log_10_POR = log_10_ *a* + *b*log_10_TL	−0.7551	0.8212	0.1953	0.0981	0.4632	0.0723	Isometry
	log_10_D1A = log_10_ *a* + *b*log_10_TL	−1.3043	1.1300	0.1361	0.0684	0.7729	0.0609	Isometry
	log_10_D1B = log_10_ *a* + *b*log_10_TL	−1.3518	1.0877	0.1954	0.0982	0.6036	0.3745	Isometry
	log_10_D1H = log_10_ *a* + *b*log_10_TL	−1.8678	1.2977	0.1762	0.0885	0.7279	0.0012	Positive allometry
	log_10_D1I = log_10_ *a* + *b*log_10_TL	−1.5744	1.0144	0.3242	0.1629	0.3207	0.9298	Isometry
	log_10_D2A = log_10_ *a* + *b*log_10_TL	−1.0716	0.8322	0.2648	0.1331	0.3227	0.2110	Isometry
	log_10_D2B = log_10_ *a* + *b*log_10_TL	−1.2704	0.9243	0.2351	0.1181	0.4295	0.5232	Isometry
	log_10_D2H = log_10_ *a* + *b*log_10_TL	−1.4886	0.8818	0.4092	0.2056	0.1787	0.5671	Isometry
	log_10_D2I = log_10_ *a* + *b*log_10_TL	−1.7979	1.0114	0.3859	0.1939	0.2468	0.9533	Isometry
	log_10_P1A = log_10_ *a* + *b*log_10_TL	−1.2687	1.2653	0.1357	0.0682	0.8111	0.0002	Positive allometry
	log_10_P1B = log_10_ *a* + *b*log_10_TL	−1.8356	1.2576	0.2485	0.1248	0.5568	0.0424	Positive allometry
	log_10_P1H = log_10_ *a* + *b*log_10_TL	−1.5858	1.3659	0.1554	0.0781	0.7923	0.0000	Positive allometry
	log_10_P1I = log_10_ *a* + *b*log_10_TL	−1.6806	1.1347	0.2028	0.1019	0.6059	0.1901	Isometry
	log_10_P2A = log_10_ *a* + *b*log_10_TL	−1.4248	1.0874	0.1745	0.0877	0.6563	0.3221	Isometry
log_10_P2H = log_10_ *a* + *b*log_10_TL	−1.4880	1.0268	0.2164	0.1087	0.5243	0.8061	Isometry

### 3.3 Fin Morphometry

Because of the quality requirements for the images used in the geometric morphometric analysis, it was only possible to proceed with a subsample of specimens: 64 individuals for the caudal fin and 26 individuals for the first dorsal fin. Specific information on the sample composition used for geometric morphometric analysis is reported in Table 5.

**TABLE 5:** Sample composition of blue sharks used for the geometric morphometric analysis of the caudal and first dorsal fins, grouped by sex (F, females; M, males) and size (A, adults; J, juveniles; SA, subadults).

		Size group	*n*	TL range (cm)	Mean TL ± SD (cm)
Caudal fin	Females	J	15	85.37–152.15	128.88 ± 18.33
		SA	13	142.78–173.54	159.50 ± 9.55
		A	7	180.37–217.71	199.76 ± 14.68
	Males	J	5	82.88–121.27	101.43 ± 16.43
		SA	7	133.89–148.48	140.07 ± 4.81
		A	17	151.15–206.77	171.72 ± 17.20
Dorsal fin	Females	J	9	103.42–132.29	120.53 ± 9.56
		SA	3	150.96–172.69	163.90 ± 11.44
		A	2	170.37–217.71	194.04 ± 33.47
	Males	J	2	105.54–169.76	137.65 ± 45.41
		SA	2	133.89–139.18	136.54 ± 3.47
		A	8	151.06–186.9	169.13 ± 12.23

Abbreviation: TL, total length.

**FIGURE 3:**
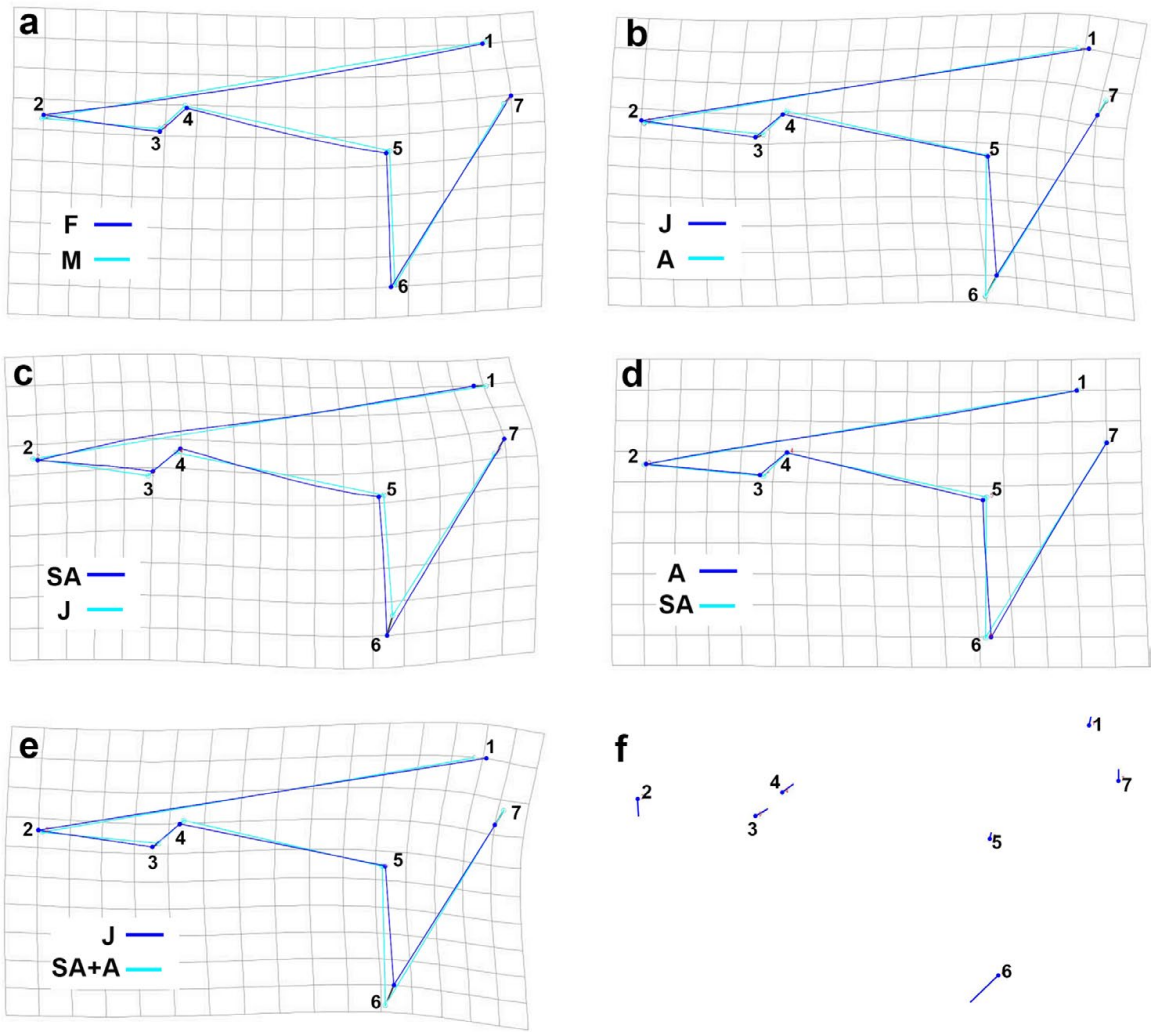
Discriminant function analysis wireframe graphs produced for *Prionace glauca* caudal fins comparing: (a) females (F) and males (M), (b) juveniles (J) and adults (A), (c) juveniles and subadults (SA), (d) adults and subadults (SA) and (e) juveniles versus a combined group of adults and subadults (SA + A). PCA produced a shape change graph (f). The numbers indicate the landmarks' positions.

The DFA indicated no statistical differences (*p* = 0.49) in the caudal fin shape between female and male blue sharks, as also revealed by the wireframe graph, which showed a high degree of overlap between the two shapes (Figure 3a). In contrast, all paired comparisons between size groups showed significant differences in caudal fin shape (J vs. A *p* < 0.0001; J vs. SA *p* = 0.0007; A vs. SA *p* = 0.033). Wireframe graphs (Figure 3b–d) revealed that these differences were mostly associated with the ventral lobe of the caudal fin, which seems to elongate and broaden during ontogenetic development, resulting in a less heterocercal fin shape. The same pattern also emerged when comparing juveniles with a combined group of adults and subadults (Figure 3e) (discriminant function analysis *p* < 0.0001). These results were confirmed by the PCA analysis, which identified the ventral lobe of the caudal fin as the main source of variation in fin shape during ontogeny (Figure 3f). Specific PCA results between groups are reported in Data S1.

**FIGURE 4:**
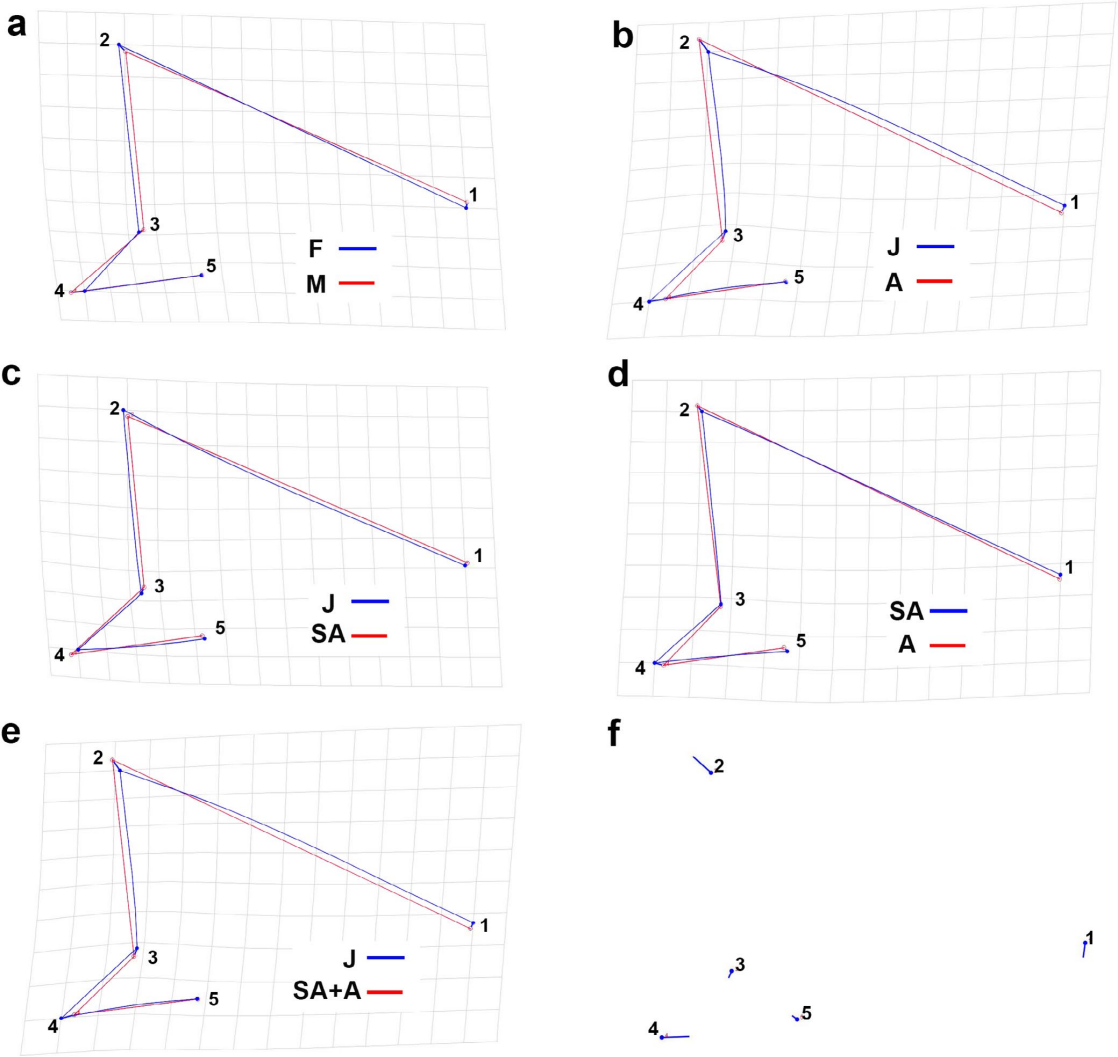
Discriminant function analysis wireframe graphs produced for *Prionace glauca* first dorsal fins comparing: (a) females (F) and males (M), (b) juveniles (J) and adults (A), (c) juveniles and subadults (SA), (d) adults and subadults, and (e) juveniles and a group of adults and subadults (SA + A). PCA produced a shape change graph (f). The numbers indicate the landmarks' positions.

Similarly to what was observed for caudal fins, the discriminant function analysis found no significant differences in dorsal fin shape between sexes (*p* = 0.29; Figure 4a). However, the same analysis indicated no statistically significant difference in all pairwise comparisons among size groups (J vs. A *p* = 0.08; J vs. SA *p* = 0.25; A vs. SA *p* = 0.89) (Figure 4b–d). In contrast, the first dorsal fin shape was found to significantly differ when comparing juveniles with a combined group of adults and subadults (discriminant function analysis *p* = 0.025). The associated wireframe graph (Figure 4e) indicated that the first dorsal fin exhibited positive allometry in height and negative allometry in the length of the posterior lobe during ontogenetic development. The shape change graph produced by the PCA seems to confirm these findings (Figure 4f). Detailed PCA outcomes for each group are provided in Data S2.

## 4 Discussion

Sharks (superorder: Selachimorpha) are ancient vertebrates encompassing over 500 species (Ebert et al. 2021). Their evolutionary success is demonstrated by their persistence over geological time (Cole and Currie 2007; Stein et al. 2018) and their ability to occupy a wide range of habitats with specialised eco‐forms (Sorenson et al. 2014; Munroe et al. 2014; Kuraku 2021; Gayford, Godfrey, and Whitehead 2023; Gayford, Whitehead, and Jaquemet 2024). Sharks exhibit morphological diversity closely tied to their habitats, showing high morphological adaptation to their environment (e.g., bottom, pelagic, meso‐ and bathypelagic) (Dulvy et al. 2014). Furthermore, sharks show a relatively high phylogenetic conservation, generating stable phenotypic forms that partly explain their evolutionary success. In other words, eco‐morphological adaptations ensure the observed evolutionary success of these animals (Sternes and Shimada 2020).

In this context, morphological studies help to identify the selective drivers that have shaped the evolutionary history of shark species (Irschick et al. 2017; Sternes and Shimada 2020; Gayford, Godfrey, and Whitehead 2023; Gayford, Whitehead, et al. 2023; Gayford, Whitehead, and Jaquemet 2024). Studies on interspecific morphological variation in relation to ecological characteristics could shed light on the evolutionary history of marine animals, including sharks (Sternes and Shimada 2020). However, the body morphology of vertebrates can also change during ontogeny, with patterns of ontogenetic variation potentially reflecting different selective pressures experienced at various developmental stages. Such patterns have been observed in several shark species (e.g., Gayford, Godfrey, and Whitehead 2023; Gayford, Whitehead, and Jaquemet 2024). Indeed, Gayford, Whitehead, et al. (2023) highlighted the potential role of environmental and/or dietary shifts as drivers of morphological variations, as evidenced by changes in the allometric growth of several shark species.

Unlike what was reported for other pelagic shark species (Gayford 2023), our results detected no signs of significant sexual dimorphism in the body proportions of blue sharks. Sex never appeared to be a significant driver to explain morphological differences in blue shark ontogenetic growth, which confirms the findings of Seamone et al. (2024). In this regard, although the blue shark exhibits marked female‐biased sexual size dimorphism (SSD; sensu Gayford and Sternes 2024), its overall body shape does not appear to differ between the sexes. This indicates that females and males display similar morphological proportions. This finding lends support to the hypothesis that the larger body size of females may be an adaptation associated with the requirements of a matrotrophic reproductive strategy. Nevertheless, future studies are needed to further investigate and confirm this interpretation.

Blue sharks are commonly considered generalist predators that feed on various taxa (Estupiñán‐Montaño et al. 2019). However, some studies have evidenced highly specialised teuthophagous behaviour in Ecuadorian waters (Loor‐Andrade et al. 2017; Rosas‐Luis et al. 2017). Nevertheless, prey preferences and diet do not substantially change throughout the life history of this species (Estupiñán‐Montaño et al. 2019), which contrasts with what has been commonly observed in other Carcharhinidae species (e.g., the bull shark; TinHan and Wells 2021). Moreover, both juvenile and adult blue sharks seem to inhabit the same areas (Vandeperre et al. 2014; Coelho et al. 2017; Zhu et al. 2023). Although spatial differentiation was seen depending on the season in the western part of the Mediterranean Sea (Poisson et al. 2024), Carbonara et al. (2023) reported the capture of specimens ranging from 123 to 207 cm in the southern Adriatic Sea during the summer. This suggests that both juveniles and adults inhabit the same areas, at least during this specific period of the year. On the basis of these data, ontogenetic changes in blue sharks are expected to be minimal, with growth of the body and fins likely to be mostly isometric. Nevertheless, Seamone et al. (2024), who initially started from the same hypothesis, found that, in addition to the expected general isometric growth pattern, several morphological variables grew allometrically in *P. glauca* . The authors explained these differences as being due to differing swimming performance needs between adults and juveniles. Indeed, although adults have a greater capacity for sustained swimming over large distances (migrations), juveniles require more burst‐like swimming, possibly to evade predators (Domenici 2001) or improve their own predatory success (Hoffmann et al. 2020).

The results of our study, on the basis of the largest sample to date, seem to confirm a significant difference between juveniles and the rest of the population (Figure 2; Table 3). Furthermore, with regard to linear morphometry, the major contributors to the differentiation between juveniles and adults/subadults appeared to be related to the overall length of the truncal region and BD. Although most of the linear measurements considered appeared to scale isometrically in juveniles (Table 4), several body regions exhibited positive allometry in the subadult and adult group (Table 4). The positive allometry observed in the distance between the snout and the second dorsal fin (PD2), the pelvic fin (PP2) and the anus (SVL), together with positive scaling of BD (Table 4), indicates that subadult and adult blue sharks tend to develop greater elongation and increased body depth in the anterior region (head and trunk) than juveniles do. Interestingly, the pectoral fins exhibited positive allometric scaling in height (P1H), anterior margin (P1A) and base length (P1B), a trend that appears to persist throughout all life stages (Table 4). This indicates that these structures continue to grow proportionally longer and wider relative to TL across the entire lifecycle.

The anterior part of pelagic sharks appears to play a crucial role in both feeding and swimming. In several shark species (e.g., the white shark *Carcharodon carcharias* , the tiger shark *Galeocerdo cuvier* , and the bull shark *Carcharhinus leucas* ), ontogenetic changes to the mouth and head (Kim et al. 2012; Fu et al. 2016; Gayford, Whitehead, and Jaquemet 2024) have been linked to shifts in diet and/or migration (Blaison et al. 2015; Fu et al. 2016; Franks et al. 2021; Rider et al. 2021). The positive allometry observed in the growth of the cephalic region of blue sharks is typically associated with the requirement to expand the prey spectrum to include larger animals in other shark species. Although the blue shark remains a generalist predator that maintains a high trophic level throughout its life (Hernández‐Aguilar et al. 2016; Meneses et al. 2016; Vidal et al. 2023), it may still be necessary for adults to expand their diet, consuming more and larger prey. This could explain the significant differences in the anterior body region and the truncal width observed across the ontogenetic groups analysed in this work. Similar differences were previously reported by Seamone et al. (2024), who found that width, length and buccal area follow a positive allometry growth scaling.

The pectoral fins of the blue shark are typically plesodic type (long and falcate with small skeletal base radials near the body) (Maia et al. 2012). Pectoral fins generally serve a manoeuvrability function in pelagic sharks (Hoffmann et al. 2019; Sternes et al. 2024; Wilga and Lauder 2002). In adults and subadults, the pectoral fins could primarily enhance hydrodynamics and facilitate longer migrations. In juveniles, the relatively larger pectoral fins could increase manoeuvrability during burst‐swimming, thereby improving predator evasion (Wilga and Lauder 2002; Sternes and Shimada 2020; Seamone et al. 2024), as well as facilitating predation in complex environments, such as coastal environments (Wilga and Lauder 2002; Sternes and Shimada 2020; Seamone et al. 2024). Juvenile blue sharks, particularly immature females, exhibit a preference for coastal habitats, using these areas for thermoregulation and to gain access to prey resources (Maxwell et al. 2019). Moreover, blue sharks (especially adults) exhibit extensive vertical movements spanning from the surface to depths exceeding 1500 m, undertaking regular diel upward and downward dives (Queiroz et al. 2012; Sternes and Shimada 2020; Vedor et al. 2021). Carbonara et al. (2024) recently demonstrated that the depth reached by blue sharks is positively correlated with their size, with larger animals able to reach greater depths. Considering the essential role of the pectoral fins in both horizontal and vertical swimming (Wilga and Lauder 2002; Fish and Shannahan 2005), it is plausible that continuous positive allometric scaling of this structure across all life stages could be linked to the varying vertical movement capabilities of blue sharks related to the size of the blue sharks (Carbonara et al. 2024).

The allometric growth of blue sharks' BD could be linked to the lower density of larger specimens compared to juveniles (Seamone et al. 2024). This lower density, which is also due to liver growth, facilitates the vertical migrations of larger specimens to depths exceeding 1000 m (Queiroz et al. 2012; Vedor et al. 2021), as well as their rapid ascent to the sea surface (Carbonara et al. 2024). This behaviour, alongside feeding (Campana et al. 2011; Rodríguez‐Cabello et al. 2016) and thermoregulation (Watanabe et al. 2021), may serve an energetic purpose (Carbonara et al. 2024). Indeed, sharks tend to have negative buoyancy and DVM could represent an energy‐saving strategy compared to continuous swimming at surface depths (Watanabe et al. 2019, 2021). From this perspective, ontogenetic morphological adaptations in BD and density may favour these daily vertical migrations (to the surface at night and to deeper waters during the day) and could explain the differences in vertical migratory capacity between small and large blue sharks (Carbonara et al. 2024). Indeed, a shift towards more neutral buoyancy in larger sharks could significantly reduce the energy required for both sustained swimming (Gleiss et al. 2017) and stronger vertical migration (Watanabe et al. 2021). In contrast, juveniles with greater negative buoyancy (i.e., smaller liver, fewer oil reserves) could benefit from increased energy availability for rapid movements (Gleiss et al. 2017), which provides them with better manoeuvrability (Seamone et al. 2024). This is particularly important in more complex and dynamic environments, such as coastal areas (Ferreira et al. 2024). Furthermore, although maintaining a generalist diet (Bazzi et al. 2021), blue sharks move away from coastal habitats as they grow older, probably shifting their feeding habits from smaller coastal fish to larger ones that typically require feeding at a wider depth spectrum in the open ocean. In this context, the need for DVM in pursuit of possible prey could be another factor affecting the shape of blue sharks throughout their development. This should be investigated further in future studies.

The geometric morphometric analysis of the caudal fin showed significant ontogenetic differences, with juveniles exhibiting a significantly more heterocercal tail than subadults and adults. Specifically, the ventral lobe of the caudal fin appeared to be significantly smaller in juveniles than in the other size classes examined (Figure 2). However, it is interesting to note that the caudal fin of these animals tends to become progressively less heterocercal throughout their life cycle. In juveniles, we observed positive allometry in the ventral lobe of the fin (CPV), which therefore appears to elongate from the earliest stages. In contrast, the dorsal lobe (CDM) grows isometrically (Table 4). Conversely, in subadults and adults, the ventral lobe (CPV) grows isometrically, whereas the dorsal lobe (DCM) even shows negative allometry (Table 4), thus contributing to reducing the heterocercality of the caudal fin. Generally, the lunate shape (homocercal and/or near‐homocercal) is considered more suited to pelagic species and thunniform swimming, whereas the heterocercal tail is characteristic of benthic species (Sternes and Shimada 2020). Maia et al. (2012) divided elasmobranchs with axial undulatory propulsion (non‐batoids) into three groups: (i) thunniform, with a high aspect‐ratio tail that is externally symmetrical for fast swimming (e.g., Lamnidae family); (ii) subcarangiform, with a lower heterocercal tail angle and wide range of swimming speeds (the blue shark is included here); and (iii) anguilliform, which comprises slow‐swimming benthic sharks with a low and highly heterocercal tail (e.g., Scyliorhinidae, Hexanchidae) (see Maia et al. 2012, figure 5.1 for further details). This narrative fits well, for example, to lamniforms (e.g., the great white shark, the mako shark, the porbeagle shark), large pelagic sharks and various species of carcharhiniforms (e.g., the leopard shark, the Blackmouth catshark). However, this distinction encompasses a continuum of intermediate shapes, and the blue shark exemplifies the fact that not all pelagic and large swimmer tails are lunate or particularly rigid (Crofts et al. 2019). Indeed, the blue shark is pelagic and its tail is neither lunate nor noticeably less flexible than other heterocercal tails. The apparent lack of adaptations in the blue shark tail for fast and sustained swimming may also be indicative of the functional trade‐offs associated with the behaviour and ecology of this species, including its DVM. The persistence of a heterocercal tail in blue sharks may serve two functions: enhancing manoeuvrability and supporting vertical migrations. In terms of manoeuvrability, lamnids have a larger turning radius than other fish with heterocercal tails, which, on the contrary, have greater manoeuvrability (Blake 2004). In any case, as discussed in previous sections, overall manoeuvrability, is the result of several morphological adaptations, including those in the pectoral fins and the shape of the cephalic region (Porter et al. 2009; Wilga and Lauder 2002). The greater heterocercality of the caudal fin in juveniles may be a response to their greater need for manoeuvrability. Indeed, a more heterocercal caudal fin can generate greater lift and propulsion, and juveniles may rely on burst swimming to escape predators. It could also be useful to provide additional lift during swimming, compensating for their lower buoyancy. Conversely, the reduced heterocercality observed in adults could be linked to the greater distances they cover and their horizontal migrations, which have been documented in both the open ocean (Campana et al. 2011) and the Mediterranean Sea (Poisson et al. 2024). Larger blue sharks, which may face less pressure from predators and display more neutral buoyancy, may benefit from caudal fin scaling to lower the cost of migration, even if it results in decreased manoeuvrability (Heithaus et al. 2007; Crofts et al. 2019; Gayford, Godfrey, and Whitehead 2023). Ontogenetic differences may also relate to differences in vertical migration on the basis of length (Carbonara et al. 2024). Larger animals, which are capable of reaching greater depths, benefit from the upward thrust guaranteed by a heterocercal tail. However, with reduced heterocercality, the tail may exert stronger propulsion; the rigidity of the tail likely also plays a fundamental role (Crofts et al. 2019).

The dorsal fin is a morphological feature that varies across shark species (Carrillo‐Aguilar et al. 2022; Sternes and Shimada 2020). It has been proven that the dorsal fin allows side‐roll lift in the great hammerhead shark ( *Sphyrna mokarran* ) in order to reduce energetic consumption (Payne et al. 2016). However, the dorsal fin primarily acts as a stabiliser, particularly during roll rotation, and may also generate secondary thrust (Lingham‐Soliar 2005; Maia and Wilga 2013). Unlike Seamone et al. (2024), our analyses did not detect any significant ontogenetic differences in dorsal fin shape when comparing juveniles, subadults and adults. However, when comparing juveniles with subadults and adults grouped together, evidence of allometric growth was observed, with the dorsal fin becoming taller (Figure 3; Table 4), similarly to what was reported by Seamone et al. (2024). Finally, it should be noted that our geometric morphometric analysis, although on the basis of a relatively small number of landmarks, successfully captured the main patterns of shape variation. However, some finer‐scale differences between groups, which would require a semi‐landmark approach, might not have been fully detected.

## 5 Conclusion

This study sheds light on the ontogenetic morphometric variation and ecomorphological adaptations of the Mediterranean blue shark population. Using the largest sample analyzed to date, we observed significant morphological differences between the juvenile and adult‐subadult groups, particularly in the head, pectoral fins, BD, and caudal fin shapes. These differences highlight the evolutionary and ecological adaptations of blue sharks to different life stages, which are likely driven by distinct functional and environmental requirements. However, despite the large sample size, the size composition of the analyzed specimens was skewed toward juveniles, most likely because of the greater difficulty in catching larger fish, particularly in the location under investigation. As a result, further research into the ontogenetic development of the Mediterranean blue shark is needed in order to rule out the possibility of bias caused by an unbalanced sample size.

Our results suggested that juvenile blue sharks exhibit morphological traits that enhance manoeuvrability and predator escape, such as a more heterocercal caudal fin. By contrast, adults and subadults display adaptations that are better suited to sustained swimming and long‐distance migrations. These adaptations include streamlined pectoral fins, an increased BD, and a less heterocercal caudal fin. These adaptations facilitate energy‐efficient vertical and horizontal movement, which are essential for accessing deeper habitats and a broader prey spectrum.

In summary, the positive allometry observed in the anterior region suggests a functional need for larger head structures in adults to broaden their prey spectrum, supporting their role as generalist predators. Meanwhile, ontogenetic changes in body and fin morphology reflect the ecological transition from predator evasion in juveniles to migratory efficiency and foraging versatility in adults and subadults.

Exploring these ecomorphological transitions provides valuable insights into the life history strategies of *P. glauca* . This research not only enhances our understanding of the ecological plasticity of this species but also underscores its critical role in the pelagic ecosystem. This paves the way for future studies that integrate genetic, physiological and behavioural dimensions to develop targeted management and conservation initiatives. Further research on other species following similar procedures is therefore recommended to improve our understanding of how sharks alter their habitat usage as they grow, which could potentially provide new information for improved management.

## Author Contributions

**P. Carbonara:** conceptualization (lead), formal analysis (equal), funding acquisition (lead), investigation (lead), project administration (lead), supervision (lead), writing – original draft (lead), writing – review and editing (lead). **A. Bellodi:** conceptualization (lead), data curation (lead), formal analysis (lead), investigation (lead), methodology (lead), software (lead), visualization (lead), writing – original draft (equal). **M. Bottaro:** formal analysis (supporting), investigation (supporting), supervision (supporting), writing – review and editing (supporting). **G. Deplano:** data curation (equal), formal analysis (equal), investigation (equal). **A. Mulas:** data curation (equal), formal analysis (equal), investigation (equal), methodology (equal), visualization (equal), writing – review and editing (supporting). **C. Neglia:** data curation (equal), formal analysis (equal), investigation (equal), methodology (equal), software (supporting), writing – review and editing (supporting). **S. Niedermüller:** funding acquisition (supporting), resources (equal), supervision (equal), writing – review and editing (supporting). **G. Prato:** funding acquisition (supporting), resources (equal), supervision (equal), writing – review and editing (supporting). **L. Toomey:** conceptualization (equal), data curation (equal), methodology (equal), supervision (equal), writing – original draft (equal), writing – review and editing (equal). **M. C. Follesa:** conceptualization (supporting), funding acquisition (supporting), investigation (equal), project administration (supporting), supervision (equal), writing – review and editing (supporting).

## Conflicts of Interest

The authors declare no conflicts of interest.

## Supporting information


**Data S1:** PCA results on the basis of *Prionace glauca* caudal fins comparing: females (F) and males (M), juveniles (J) and adults (A), juveniles and subadults (SA), adults and subadults (SA), and juveniles versus a combined group of adults and subadults (SA + A).

**Data S2:** PCA results on the basis of *Prionace glauca* first dorsal fin comparing: females (F) and males (M), juveniles (J) and adults (A), juveniles and subadults (SA), adults and subadults (SA), and juveniles versus a combined group of adults and subadults (SA + A).

**Table S1:** Linear relationship parameters (a, b) and the relative *R* ^2^ for every measurement recorded in *Prionace glauca* females and males. The relative graphs are reported aside.

**Table S2:** SIMPER ANALYSIS results for blue sharks juveniles (J) against the combined adult and sub‐adult group (SA + A).

## Data Availability

All the required data are uploaded as Supporting Information.
